# PIK3CA alterations in Middle Eastern ovarian cancers

**DOI:** 10.1186/1476-4598-8-51

**Published:** 2009-07-28

**Authors:** Jehad Abubaker, Prashant Bavi, Wael Al-Haqawi, Zeenath Jehan, Adnan Munkarah, Shahab Uddin, Khawla S Al-Kuraya

**Affiliations:** 1 Department of Human Cancer Genomic Research, Research Center, King Faisal Specialist Hospital and Research Center, Riyadh, Saudi Arabia; 2 Department of Obstetrics and Gynecology, King Faisal Specialist Hospital and Research Center, PO Box 3354, Riyadh 11211, Riyadh, Saudi Arabia

## Abstract

**Background:**

PI3K/AKTsignaling pathway plays an important role in cell growth, proliferation, and tumorgenesis of various malignancies. This signaling pathway has been shown to be frequently altered in several human cancers including ovarian cancers. However the role of this oncogenic signaling pathway has not been explored in the Middle Eastern epithelial ovarian cancer (EOC). Therefore, we investigated PI3K/AKT genetic alterations such as PIK3CA amplification, PIK3CA mutation, PTEN protein loss and their relationships with various clinicopathological characteristics in 156 EOCs.

**Results:**

Fluorescence *in situ *hybridization (FISH) technique and DNA sequencing were used to analyze PIK3CA amplification and mutation respectively. Expression of PIK3CA protein expression (p110 α), PTEN, p-AKT and Ki-67 was analyzed by immunohistochemistry. *PIK3CA *amplification was seen in 54 of 152 (35.5%) EOC cases analyzed; PIK3CA gene mutations in 6/153 EOC (3.9%); *KRAS *mutations in 3/154 EOC (1.9%), BRAF mutations in 3/156 EOC (1.9%), p53 mutation in 50/154 EOC (32.5%), and loss of PTEN protein expression in 33/144 EOC (22.9%). p110 α overexpression was associated with increased phosphorylation of AKT-Ser 473 and with the proliferation marker Ki-67.

**Conclusion:**

Our data showed mutual exclusivity between the molecular event of PIK3CA amplification and mutations in *PIK3CA*, *KRAS*, *BRAF *genes, which suggests that each of these alterations may individually be sufficient to drive ovarian tumor pathogenesis independently. High prevalence of genetic alterations in PI3K/AKT pathway in a Middle Eastern ovarian carcinoma provides genetic evidence supporting the notion that dysregulated PI3K/AKT pathways play an important role in the pathogenesis of ovarian cancers.

## Background

Ovarian carcinoma is the most lethal gynecological malignancy because most tumours are detected in advanced stages [1,2]. It is the fifth leading cause of cancer-related death among U.S. women. In Middle Eastern ovarian cancer, a similar pattern of incidence has been seen [3]. Epithelial cancer of the ovary derives from malignant transformation of the epithelium of the ovarian surface, which is contiguous with the peritoneal mesothelium [4,5]. Epithelial ovarian neoplasms are subclassified histologically into serous, mucinous, endometrioid, clear cell, transitional (Brenner), squamous cell, and undifferentiated subtypes. Serous carcinomas (SC) are the most common histology, accounting for about two thirds of ovarian carcinomas [6]. A better understanding of the factors and mechanisms determining the aggressive behavior of some EOC is critical in developing new treatment.

Increased mitogenic signaling through receptors has proven to play a major role in ovarian cancer. One of the major downstream mediators of signaling initiated by these receptors is the phosphatidylinositol 3-kinase (PI3K)/AKT pathway. PI3K/AKT is activated in multiple cancers leading to oncogenic transformation [7,8]. Activation may result from activating mutation of the catalytic subunit of p110α of PIK gene (PIK3CA) or amplification of PIK3CA or as a result of inactivating mutation in the tumor suppressor gene, phosphatase and tensin homolog (PTEN). Activation of this pathway will cause AKT to translocate to the plasma membrane, where it is activated by phosphorylation allowing it to mediate many of the biological consequences of PI3K activation. Several studies have showed PIK3CA gene amplification in ovarian cancers [9-13]. Mutations in the *PIK3CA *have also been identified in ovarian cancers and though relatively common in endometrioid ovarian carcinomas (EC), are uncommon in serous carcinomas (SC) [11].

Rat sarcoma viral oncogene (RAS) proteins are located on the inner surface of the plasma membrane and are attached to the membrane by a farnesyl residue. RAS proteins transmit extracellular signals that promote the growth, proliferation, differentiation and survival of cells. The major downstream target of RAS-GTP is mitogen-activated protein kinases (MAPKs), but it is also known to activate other targets like PI3K [14,15]. The MAPK pathway is hyperactivated in 30% of human cancer [14]. RAS mutation can promote ovarian tumorigenesis through MAPK pathway or through its interaction with PI3K/AKT pathway [14,15]. In ovarian cancer, KRAS mutation has been identified in 35% low-grade serous tumors and 33% of borderline tumors, whereas, BRAF mutations occur in 30% of low-grade serous carcinomas and 28% of borderline tumors [16,17]. KRAS and BRAF mutations are less common in high grade ovarian cancers [16,17].

Several pervious studies have shown the oncogenic role of PI3K/AKT pathway in EOC. Elucidation of the characteristics of ovarian cancer with PI3K alterations seems to be important for the execution of personalized medicine in future. However, all the studies on PI3K alteration reported until now dealt with Caucasian ovarian cancers. It seems interesting to compare the frequency of PI3K alterations between two ethnicities, Middle Eastern and Caucasian EOC, and explore the contribution of PI3K alterations to pathogenesis of Middle Eastern EOC. Therefore, in the present study, we have analyzed the prevalence of genetic alterations of PI3K in Middle Eastern EOC and their interrelationships with other genetic alterations like p53, KRAS and BRAF. Furthermore, correlation of the PI3K alterations with various clinicopathological characteristics was investigated in a large cohort of Middle Eastern EOCs.

## Methods

### Patient selection and tissue microarray construction

156 patients with ovarian carcinoma diagnosed between 1991–2007 were selected from the files of the King Faisal Specialist Hospital and Research Centre. All samples were analyzed in a tissue microarray (TMA) format. TMA construction was performed as described earlier [18,19]. Two cores of ovarian carcinoma were arrayed from each case. The Institutional Review Board of the King Faisal Specialist Hospital & Research Centre approved the study.

The patients were diagnosed histologically and received follow-up care in the Departments of Obstetrics and Gynecology and Oncology at King Faisal Specialist Hospital and Research Centre. The histological subtype of each ovarian tumor sample was determined by pathologist (PB) according to accepted criteria [20]. Department of Obstetrics and Gynecology, King Faisal Specialist Hospital and Research Center provided long-term follow-up data for these patients. The primary pathological diagnosis was serous 125 patients (80.1%), endometrioid in 22 (14.1%), clear cell in 4 (2.6%) and undifferentiated/mixed Epithelial in 5 (3.2%). The ages of the patients ranged from 19–86 years, with a median age of 56 years. The majority of patients underwent primary surgical staging or cytoreduction. In some patients who were not fit for primary surgery, primary neoadjuvant chemotherapy was followed by interval debulking surgery. The distribution by FIGO stage at diagnosis was: stage I-II in 8 patients (5.1%), stage III-IV in 137 (87.8%), and unknown in 11(6.1%). The median follow-up time was 14.9 months (range, 2–130 months). Progression free survival was computed from date of surgery for patients who underwent primary cytoreduction and from date of diagnosis by biopsy or cytology in those who underwent primary neoadjuvant chemotherapy. Since the majority of patients are lost to follow-up as their disease reaches its terminal stages, it was impossible to determine overall survival in this specific patient population.

### DNA extraction and purification

Genomic DNAs were extracted from paraffin embedded neoplastic primary tissues using Gentra Kit (Minneapolis, MN, USA) following a slight modification to the manufacturer's recommendation.

### Mutation analysis of PIK3CA, p53, KRAS, and BRAF genes

Step-down cycling condition was used for BRAF T1799A transversion mutation in exon 15 of the BRAF gene [21]. After a 10-min denaturing at 95°C, the PCR was run with each temperature for 1 min at five step-down steps, for two cycles each. The denaturing temperature was 95°C, and extension temperature was 72°C for each step, with the annealing temperature of 66, 64, 62, 60 and 58°C from the first to the last step. The PCR was finally run at 95, 58, and 72°C each for 1 min for 35 cycles, followed by an elongation at 72°C for 5 min. PCR was performed in a total volume of 25 μL using 50 ng of genomic DNA, 2.5 μL 10 × Taq buffer, 1.5 μL MgCl2 (25 mM), 0.05 μL dNTP (10 mM), 0.2 *μL *Taq polymerase (1 U/*μ*L) (all reagents were from Qiagen Inc), 1 μL of each primer (2.5 *μ*M), and water. As majority of KRAS mutations were found in exon 1 of *KRAS *gene, we focused our mutation analysis on *KRAS*.[21] The PCR mixture contained the same components as in PCR reaction for the *BRAF *gene. The PCR condition was as follows: after a 10 min denaturation at 95°C, 30 s of annealing at 53°C, and 1 min of extension at 72°C, with an extension of 72°C for 7 min at the last step [21]. The efficiency and quality of the amplification PCR were confirmed by running the PCR products on a 2% agarose gel. The PCR products were subsequently subjected to direct sequencing PCR with BigDye terminator V 3.0 cycle sequencing reagents (Applied Biosystems, Foster City, CA). The samples were finally analyzed on an ABI PRISM 3100 xl Genetic Analyzer (Applied Biosystems, Foster City, CA). Sequencing of *PIK3CA *exons 9, 20 was done by PCR amplification and direct sequencing of both strands for all CRC cases and their matched normal samples as previously described [22,23].

For *p53 *mutational analysis, exons 5–8 of the *P53 *gene were amplified separately as previously [24]. The following primers were used: exon-5-forward: 5'GACTTTCAACTC-TGTCTC3', reverse: 5'CTGGGGACCCCTGGGCAAC3'; exon-6-forward: 5'GAGAC-GACAGGGCTGGTT3', reverse: 5'CCACTGAC-AACCACCCTT3'; exon-7-forward 5'CCAAGGCGCACTGGCCTC3', reverse: 5'GCGGCAAGCAGAGGCTGG3'; and exon-8-forward: 'CCTTACTG-CCTCTTGCTT3', reverse 5'TGAATCTGAGGCATAA-CTGC3'. The samples were finally analyzed on an ABI PRISM 3100 xl Genetic Analyzer (Applied Biosystems, Foster City, CA). Mutational analysis was done using DNA SEQMAN software (DNASTAR Inc., Madison, WI).

### Immunohistochemistry (IHC)

TMA slides were processed and stained manually. The streptavidin-biotin peroxidase technique with diaminobenzidine as chromogen was applied. For antigen retrieval, Dako Target Retrieval Solution pH 9.0 (Catalogue number S2368) was used, and the slides were boiled in a pressure cooker (Pascal Pressure Cooker, Dako Cytomation, Model: S2800, USA). Primary antibodies used, their dilutions, and incidences are listed in Table 1. Endogenous peroxidase activity was quenched using 3% hydrogen peroxidase. Endogenous biotin was blocked and all slides were counterstained with hematoxylin, dehydrated, cleared, and cover slipped with premount. Only fresh cut slides were stained simultaneously to minimize the influence of slide ageing and maximize repeatability and reproducibility of the experiment.

**Table 1:** Antibodies used for tissue microarray Immunohistochemical analysis

**Antibody (Subcellular Localization)**	**$Positive cases (%)**	**Clone**	**Company**	**Source**	**Dilution Overnight in incubation**	**Retrieval**	**Detection System**
PTEN^@^	111/144 77.1%	6H2-1	Cascade Bioscience	Mouse monoclonal	1:100	pH 9, Pressure Cooker	EnVision+
p-AKT (Cytoplasmic & Nuclear)	75/144 52.1	Ser473	Cell Signaling	Mouse Mono-clonal	Predilute	pH 9, microwave	Survival Marker; Signal Stain IHC detection Kit
PIK3CA-110	78/140 55.7%	1G12E9	Everest Biotech	Goat Polyclonal	1:400	pH 6, Pressure Cooker	EnVision+
P53	68/138 49.3%	DO-7	D9K0	Mouse monoclonal	1:50	pH 9, microwave	Ventana Bechmark

@Normal expression of PTEN was seen in 77.1% cases and PTEN inactivation (loss/reduced expression) was seen in 33/144(22.9%) of EOC cases.

$ Non representative spots for the various antibodies ranged from18 cases for p53 to 12 spots for PTEN IHC

p-AKT scoring was done as described earlier [21,25]. For purposes of statistical analysis, all cases staining at level 0 or 1 were grouped as p-AKT negative and all cases staining at level 2 and level 3 were grouped as p-AKT positive. For PTEN scoring cases staining at level 2 or 3 were considered as normal expression and cases staining at level 0 or 1 were considered to have PTEN inactivation. Only fresh cut slides were stained simultaneously to minimize the influence of slide ageing and maximize repeatability and reproducibility of the experiment. Two types of negative controls were used for p-AKT. One was the negative control in the kit in which the primary antibody was omitted. A preabsorption experiment using p-AKT Ser 473 blocking peptide (Cell Signaling Technology, Beverly, MA, Product No 1140) was used as the second negative control. PIK3CA protein (p110 α expression) was categorized by doing an H score as described earlier [26]. Each TMA spot was assigned an intensity score from 0–3(I_0_, I_1–3_) and proportion of the tumor staining for that intensity was recorded as 5% increments from a range of 0–100(P_0_, P_1–3_). A final H score (range 0–300) was obtained by adding the sum of scores obtained for each intensity and proportion of area stained (H score = I_1X_P_1_+I_2_XP_2_+I_3_XP_3_). Ovarian tumors were grouped into 2 groups using X-tile bioinformatics software: low p110 α expression (H score = 100) and the other group showed high p110 α expression (H score >100) [27].

### Flourescent in situ hybridization (FISH) methodology

FISH on tissue micorarray was performed as previously described [21]. Briefly the search for FISH probe was done by browsing Ensemble Genome Browser  for bacterial artificial chromosome (BAC) corresponding to *PIK3CA *gene. BAC RP11-245 C23 was purchased from Childrens Hospital Oakland Reseach Institute (Oakland, California), was cultured and DNA isolated. BAC DNA probe was labeled with digoxigenin using the DIG-nick translating kit from Roche. FISH was performed with a digoxigenin-labeled BAC DNA probe, containing the *PIK3CA *gene and a Spectrum Orange-labeled chromosome 3 centromeric probe (CEP3) as a reference (purchased from Vysis). TMA sections were treated according to the Paraffin Pretreatment Reagent Kit protocol (Vysis, IL, USA) before hybridization. For the ovarian cancer TMA study, hybridization and post-hybridization washes were according to the Vysis LSI procedure. Probe visualization using fluorescent isothiocyanate (FITC)-conjugated sheep anti-digoxigenin (Roche Diagnostics, Indianapolis, IN, USA) was as described (Wagner et al). Slides were then counterstained with 125 ng/ml 4',6-diamino-2-phenylindole in an antifade solution and screened with a Olympus BX51 fluorescent microscope. Tissue samples were classified with a PIK3CA/centromere 3 ratio of 1.0 as normal, between 1.0 and 2.0 as having PIK3CA gains. A PIK3CA/centromere 3 ratio of more than 2.0 was considered as amplified.

### Quantitative real-time PCR

Ovarian tumors with increased copy number by FISH of PIK3CA gene were selected for validation by quantitative real time PCR. DNA content was normalized to that of long interspersed elements (LINE1), a repetitive element for which copy number per haploid genome are similar both in normal DNA sample and neoplastic cells. Primers were designed by Primer express 3.0 software (Applied Biosystems Foster City CA) hybridize to sequences of genomic DNA for *PIK3CA *and *LINE 1*. Primers to genomic sequences were (PIK3CA forward, 5'-TATGGTTGTC-TGTCAATCGGTGA-3'; reverse,5'-GCCTTTGCAGTGAATT-TGCAT-3') and (LINE1 forward, 5'-CCGCTCAACTACATGGAAACTG-3' reverse, 5'-GCGTCCCAGAGATTCTGGTATG-3'). Conditions for all PCRs were optimized in gradient cycler (MJ Research, MA, United States) with regard to *Taq *DNA polymerase, forward and reverse primers, MgCl_2 _concentrations, dNTP concentrations and various annealing temperatures (55–65°C). Specificity of the PCR product was confirmed by agarose gel electrophoresis. Optimized results were transferred on the following Light Cycler PCR protocol.

All reactions were performed in glass capillaries (Roche, Mannheim, Germany) with a final reaction volume of 10 μl of 1× LightCycler-FastStart DNA Master SYBR Green I reaction mixture (Roche, Mannheim, Germany) containing FastStart *Taq*, reaction buffer, and deoxynucleoside triphosphate, 1 mM MgCl_2_, and final concentrations of 0.5 μM for each primer. MgCl_2 _concentrations were optimized for each target gene (varied from 2–4 mM). Thermocycling and detection were performed on the LightCycler (Roche Diagnostics, Mannheim, Germany). An initial preheating step of 10 min at 95°C was used to activate the DNA polymerase, then, a touch-down procedure, consisting of 10 s at 95°C, annealing for 5 s at temperatures decreasing from 63 to 59°C, and ending with an extension step at 72°C for 10 s. A total of 45 cycles were performed, followed by melting curve program (60–95°C with a heating rate of 0.1°C per second and a continuous fluorescence measurement), and finally a cooling step to 40°C.

Pfaffle method for relative quantification was used to calculate fold of changes for normal and ovarian cancer samples [28]. The relative copy number ratio of a target gene is calculated based on efficiency (E) and crossing point (CP) deviation of samples (normal) versus (ovarian tumor), and expressed in comparison to a reference gene (LINE1). For a normal cell the copy number of a gene per haploid genome should be one.

### Statistics

All Statistical analysis will be performed using the Statview JMP software (version 7.0). Fisher's exact chi-square (χ^2^) test was used to assess associations between categorical variables. Kaplan-Meier survival analyses were carried out for progression free survival, using the log-rank test for differences between groups. Results were considered statistically significant when p from a two-tailed test was < 0.05.

## Results

### PIK3CA mutations, PIK3CA amplification and PIK3CA 110 alpha subunit protein expression (p110 α expression) in EOC

**Figure 1:**
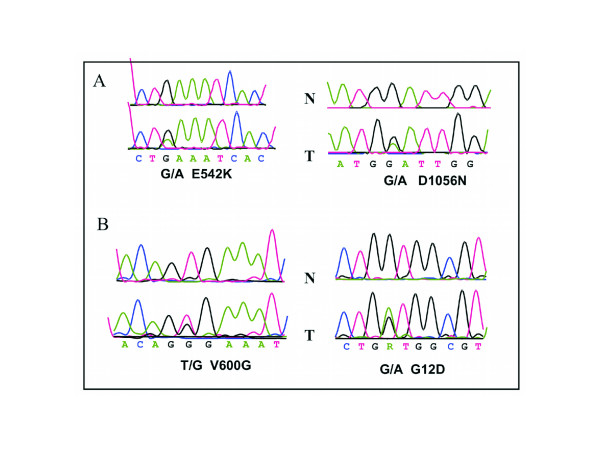
**Examples of PIK3CA, KRAS, and BRAF mutations in EOC cases**. A. Typical sequencing traces of PIK3CA mutations are shown for exon 9 (right) and 20 (left) with cancer mutant sequence (bottom) and normal wild-type sequence (top). B. Sequencing traces of BRAF (right) and KRAS (left) mutations with cancer mutant sequence (bottom) and normal wild-type sequence (top). Arrows denote position of the missense mutations with amino acid changes noted.

**Figure 2:**
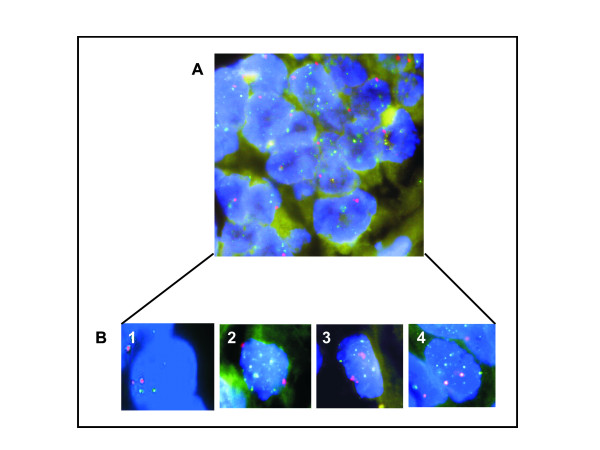
**Determination of PIK3CA gene copy number by FISH on ovarian tissue (A)**. B. Sample 1 is normal cell selected according to FISH analysis. Samples 2–4 are PIK3CA amplified ovarian tumor cells selected according to FISH. FISH images show cell nuclei (blue) from selected cases, hybridized with probes directed against PIK3CA gene (green RP11-245 C23) and centromere 3 (red). **(1)**. Normal cell (blue) shows 2 centromeric signals (red) and 2 (green) PIK3CA signals. **(2–4)**. Representative cells show amplification two (red) centromeric signals and green PIK3CA amplified Signals.

**Figure 3:**
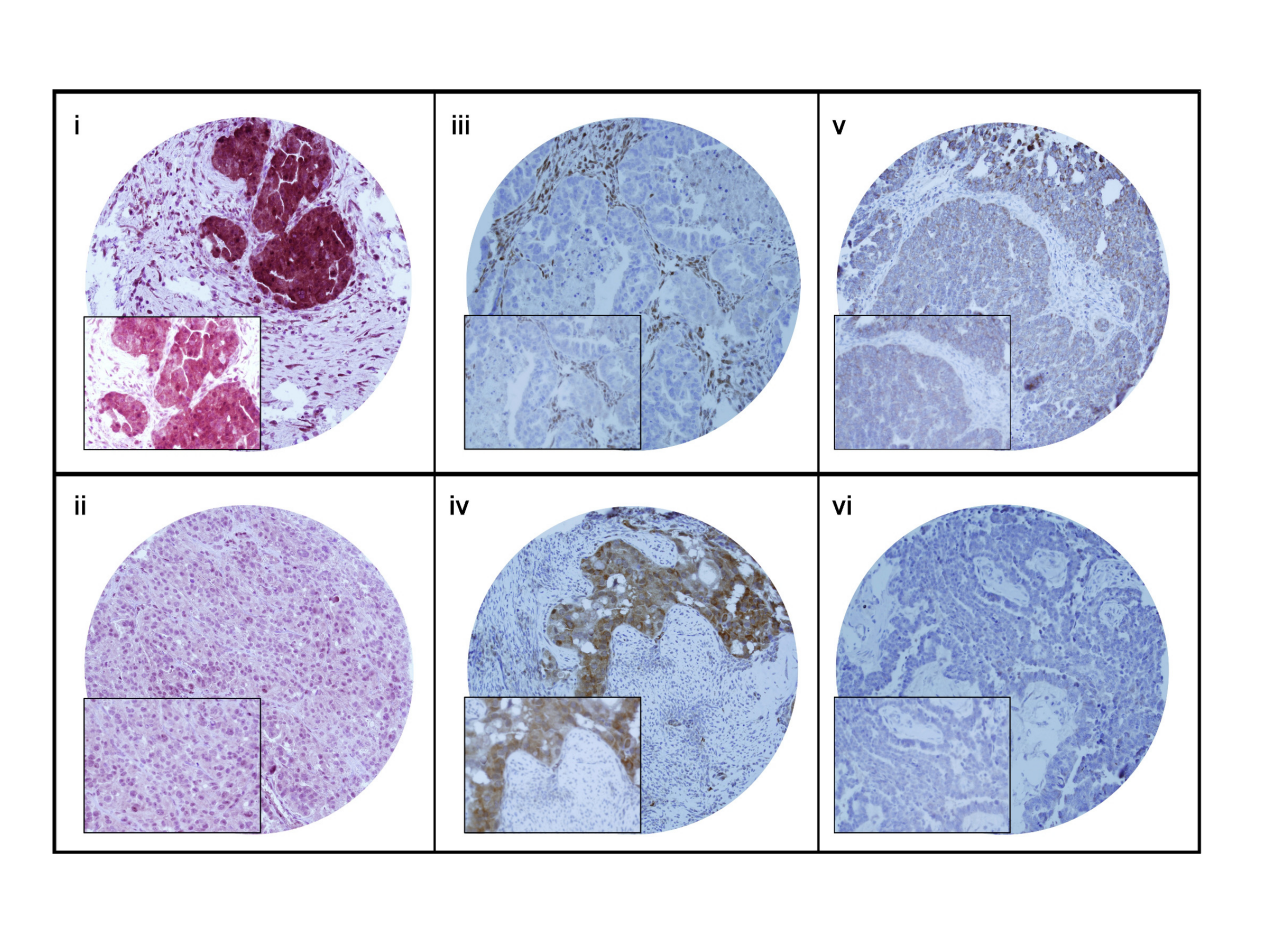
**Immunohistochemical analysis of p-AKT, PI3K-110 alpha subunit protein expression and PTEN in epithelial ovarian carcinoma (EOC)**. (i) p-AKT over expression was observed along with (iii) low PTEN expressionand (v) over expression of PI3K-110 alpha in EOC TMA specimen and, (ii) Low expression for p-AKT was seen along with (iv) high PTEN expression and (vi) reduced expression for PI3K-110 alpha in EOC TMA specimen. 20 × magnifications with the inset showing a 100 × magnified view of the same.

PIK3CA mutation was seen in 6 of 153 (3.9%, Fig. 1) EOC's analyzed and FISH analysis revealed the presence of PIK3CA amplification in 54 of 152 (35.5%, Fig. 2) EOC cases analyzed. PIK3CA amplification results were further validated by real-time PCR. No significant associations were observed with PIK3CA mutations or PIK3CA amplifications and clinicopathological parameters. Immunohistochemical analysis showed overexpression of p110 α expression in 78 of 140 (55.7%, Fig. 3) EOC cases analyzed and its association with various clinicopathological parameters was analyzed (Table 2). p110 α expression overexpression did not coorelate with age, tumor stage, FIGO grade and progreesion free survival. PIK3CA 110 overexpression was associated with overexpression of p-AKT-Ser 473 (*p *= 0.0260) and a trend was noted with the proliferation marker Ki-67 (*p *= 0.0639). Of the 6 EOCS with PIK3CA mutation, 5 of them showed overexpression of PIK3CA 110 protein. No association was seen with PIK3CA amplification and overexpression of p110 α expression (*p *= 0.2320).

**Table 2:** Correlation between PIK3CA-110-Alpha status and clinicopathological features in Epithelial Ovarian Carcinoma

			**High p110 α expression**		**Low p110 α expression**		**P value**
				
	**N**	**%**	**N**	**%**	**N**	**%**	
**Total Number of Cases**	140		78	55.7	62	44.3	

**Age**							
< = 50 years	54	38.6	31	57.4	23	42.6	0.7491
> 50 years	86	61.4	47	54.7	39	45.3	

**Tumour Stage**							
Stage I-II	8	6.1	4	50.0	4	50.0	0.7525
Stage III-IV	122	93.9	68	55.7	54	44.3	

**Histopathology**							
Clear cell	4	2.9	3	75.0	1	25.0	0.8585
Endometriod	19	13.6	11	57.9	8	42.1	
Serous	113	80.7	62	54.9	51	45.1	
Undifferentiated	4	2.9	2	50.0	2	50.0	

**FIGO Grade**							
Well differentiated	27	19.3	12	44.4	15	55.6	0.4114
Moderately Diff	73	52.1	42	57.5	31	42.5	
Poorly Diff	40	28.6	24	60.0	16	40.0	

**PAKT (Ser473)**							
High (2–3)	69	51.5	46	66.7	23	33.3	0.0260
Low (0–1)	65	48.5	31	47.7	34	52.3	

**PTEN**							
Low (0–1)	31	23.3	17	54.8	14	45.2	0.7675
High (2–3)	102	76.7	59	57.8	43	42.2	

**Ki-67**							
Above 50	51	37.0	34	66.7	17	33.3	0.0639
Below = 50	87	63.0	44	50.6	43	49.4	

**P53**							
Negative	65	50.0	37	56.9	28	43.1	0.5923
Positive	65	50.0	40	61.5	25	38.5	

**KRAS Mutation**							
Present	2	1.4	0	0.0	2	100.0	0.0720
Absent	136	98.6	76	55.9	60	44.1	

**BRAF Mutation**							
Present	3	2.1	1	33.3	2	66.7	0.4302
Absent	137	97.9	77	56.2	60	43.8	

**PIK3CA Mutation**							
Present	6	4.4	5	83.3	1	16.7	0.1298
Absent	131	95.6	70	53.4	61	46.6	

**P53 Mutation**							
Present	47	34.1	25	53.2	22	46.8	0.7497
Absent	91	65.9	51	56.0	40	44.0	

**PIK3CA FISH**							
Amplified	49	35.5	24	49.0	25	51.0	0.2320
Non-Amplified	89	64.5	53	59.6	36	40.4	

PFS-Median (months)			16.2		17.1		0.6578

### KRAS, BRAF, and TP53 mutation analysis

We found that six EOCs contained PIK3CA mutation, three EOC contained activating *KRAS *mutations and three EOCs had *BRAF *mutations at codon 599 (Fig. 1). The presence of *KRAS *and *BRAF *mutations was mutually exclusive. EOCs with PI3K mutation did not display BRAF or KRAS mutations. Thus PIK3CA mutations were also mutually exclusive with KRAS and BRAF mutation.

TP53 mutation analysis revealed a mutation incidence rate of 32.5% (50 of 154). No significant associations were found between TP53 mutations and clinicopathological data like FIGO staging, tumor grading, tumor type, patient age and progression free survival. Also TP53 mutations were not associated with mutations in PIK3CA, BRAF, and KRAS genes or with activation of AKT, loss of PTEN expression and PIK3CA amplification.

Using the mouse monoclonal anti p53 antibody (Table 1), p53 overexpression by immunohistochemistry was seen in 68/138 (49.3%) EOCs analyzed. p53 overexpression was significantly associated with p53 mutations (*P *= 0.0002) and a trend of increased expression noted in poorly differentiated EOCs (*P *= 0.0961).

### AKT activation in EOC and its correlation with PIK3CA mutations, PIK3CA amplification, KRAS, BRAF mutations

Because it is suggested that PIK3CA mutations activate AKT function through its phosphorylation [29], we investigated the relationship between PIK3CA mutation, PIk3CA amplification and activation of p-AKT. The correlation between the activation of AKT with clinicopathological parameters and mutational status of *K-KRAS and BRAF *genes and *TP53 *mutation was also explored. AKT activation was evaluated by assessing phosphorylation of AKT by immunohistochemistry at Ser473 and an EOC case was considered to show AKT activation when it was scored as 2+ or 3+ for p-AKT Ser 473. Activation of AKT was observed in 52.1% (75 of 144) of EOC cases.

Though p-AKT Ser 473 overexpression correlated significantly with proliferation marker, Ki-67 expression (*p *= 0.0262), no association was seen with clinicopathological parameters including progression free survival. Also there was a lack of association between EOCs that harbored *KRAS *mutations or *BRAF *mutations and AKT activation.

### PTEN inactivation

All the EOC cases with lost or reduced PTEN expression were considered as EOCs with PTEN inactivation. PTEN inactivation was seen in 33 of 144 (22.9%) EOC's analyzed and was associated with histology subtype of clear cell and undifferentiated carcinomas (*p *= 0.0491); overexpression of p53 protein (*p *= .0413) and a trend was seen with older age (*p *= 0.0936). No significant associations were noted with clinicopathological features and the various mutations analyzed.

### Mutual exclusivity among the genetic alterations in the PI3K/Akt and MAPK pathways in Middle Eastern EOC cases

Because *PIK3CA *copy gain is the most common genetic alteration in the PI3K/Akt pathway in EOCs in this Middle Eastern cohort, we analyzed its relationship with each of the gene mutations in the PI3K/Akt pathway. As shown in Table 3, *PIK3CA *gene copy gain was uncommonly overlapped with gene mutations in EOC; mutations were mostly seen in the group of EOC without *PIK3CA *copy gain. The mutual exclusivity between *PIK3CA *copy gain and any of the *PIK3CA*, *KRAS*, and *BRAF *mutations was not statistically significant, probably due to the small number of each of these mutations. There was not a single overlap among these alterations. Additionally, the majority of the amplified PIK3CA cases harbored normal PTEN protein expression. However, overlap between the PTEN protein loss and *PIK3A *amplification was seen in a number of cases of EOC (5.7% [4/140]; data not shown).

**Table 3:** Summary of individual cases of EOC with genetic alterations in PIK3CA, BRAF and KRAS genes

	**KRAS Mutation**				**PIK3CA Mutation**			**BRAF Mutation**		
			
**Case number**	**Exon/codon**	**Nucleotide exchange**	**Amino acid**	**PIK3CA amplify**	**Exon/codon**	**Nucleotide exchange**	**Amino acid**	**Exon/codon**	**Nucleotide exchange**	**Amino acid**
	
1				Normal	20/1047	C**A**T>C**G**T	His>Arg			
2				Normal	20/1056	**G**AT>**A**AT	Asp>Asn			
3				Normal	20/1115	T**C**T>T**T**T	Ser>Phe			
4				Normal	9/542	**G**AA>**A**AA	Glu>Lys			
5				Normal	9/545	**G**AG>**A**AG	Glu>Lys			
6				Normal	9/530	C**A**G>C**G**G	Gln>Arg			
7	1/12	G**G**T>G**T**T	GLy>Val	Normal						
8	1/13	G**G**C>G**A**C	GLy>Asp	Normal						
9	1/12	G**G**T>G**A**T	GLy>Asp	Normal						
10				Normal				15/594	G**G**T>G**A**T	Asp>Gly
11				Normal				15/600	G**T**G>G**G**G	Val>Gly
12				Normal				15/600	G**T**G>G**A**G	Val>Glu
13				Amplified						
14				Amplified						
15				Amplified						

## Discussion

Similarities in the prevalence of *PI3K *alterations in Western and Asian ovarian cancer [29-32], have raised an interest to study these alterations in other ethnic groups. There is a mixed report on the incidence of PIK3CA mutation in ovarian cancer. Reported incidence of PIK3CA mutation in epithelial ovarian cancer varies from 3.6% [30] to 12% [31]. Our study showed a lower incidence of *PIK3CA *mutations (3.9%), indicating that the *PIK3CA *gene mutation is not a common mechanism in the activation of PIK3CA in Middle Eastern ovarian tumors. Earlier studies have reported PIK3CA gene amplification to be in the range of 13 to 24.5% [10-13,33]. Only one study done on a limited number of ovarian samples (n = 12) has reported a higher incidence of PIK3CA amplification (40%) [9]. On the other hand, our study demonstrated *PIK3CA *amplification in ovarian tumors, with a relatively high frequency (35.5%, Fig. 2), which suggests the major role of PIK3CA amplification in the activation of these ovarian cancers. Interestingly, there was an almost perfect reciprocal association of the presence of gene amplification and a somatic *PIK3CA *mutations suggesting that these mutations mainly occur in tumors without amplification. In agreement with an earlier report by Woenckhaus J et al., 2007 we also did not find any association between PIK3CA amplification and p110 α protein expression [13]. This lack of association could be explained by transcriptional or post transcriptional mechanisms which finely tune the expression level of p110 α expression. However, we found p110 α expression to be correlated with p-AKT (*P *= 0.0260, Table 2) and a trend was noted with the proliferation marker Ki-67 (*P *= 0.0639).

Next, PTEN protein expression was investigated by immunohistochemistry and 33 of 144 (22.9%, Fig. 3) EOCs were showing PTEN inactivation, which was in concordance with early report [34]. Correlation analysis between *PIK3CA *amplification and *PTEN *protein loss by IHC revealed that the majority of *PIK3CA *amplifications were found in cases with high *PTEN *protein expression. This data suggests that a single oncogenic alteration along this pathway is sufficient to drive ovarian cell transformation. As a read out of PI3K functional activation, we tested AKT phosphorylation (activation) in EOC. Our findings show that AKT is activated in a large proportion of EOCs (52.1%), which reflect the activity of that pathway in our EOC. However, no significant association was found between AKT and PIK3CA mutation/amplification. Currently, multiple pathways have been implicated as having role in AKT activation such as MAP kinase [35]. Furthermore, in agreement with an earlier report [36], we have found significant correlation between fatty acid synthase (FAS) protein expression and AKT activation in Middle Eastern EOC (data not shown). Therefore, FAS overexpression might be one of the signaling pathways to activate AKT by a mechanism independent of the PI3K pathway.

We were also interested in studying the relationship between PI3K/AKT pathway and *p53 *(tumor suppressor gene) since interactions between the *p53 *and *PI3K/AKT *pathways play a significant role in the determination of cell death/survival. Interrelation between these pathways occurs through the transcriptional regulation of *PTEN and PIK3CA *by *p53*, which is required for *p53*-mediated apoptosis [37-39]. *P53 *gene mutation is among the frequent genetic alteration in ovarian carcinomas and its incidence in Middle Eastern EOCs is 32%, which is relatively lower to what has been reported in the west (50 to 80%) [40-42]. Recently, Astanehe et al., 2008 have suggested that p53 inactivation results in increases of PIK3CA transcripts beyond those that result from amplification of PIK3CA alone [37]. Thus, the combined effects of PIK3CA amplification and p53-mediated regulation of PIK3CA and PTEN expression should contribute greatly to the increased signaling through PI3K pathway in Middle Eastern EOC.

We also analyzed MAPK signaling pathway by investigating the mutational status of key genes of that pathway, BRAF and KRAS. Presence of KRAS and BRAF mutations was significantly more common in serous borderline ovarian tumors as compared to serous carcinomas (*p *= 0.0221; KRAS mutations) and (*p *< 0.0001; BRAF mutations); data not shown). This is in concordance with earlier reports [16,17]. The mutual exclusivity of BRAF and KRAS mutations (Table 3) in our ovarian carcinoma is consistent with previous findings in ovarian carcinoma, melanoma, and colorectal carcinoma [43-45]. This also supports the view that BRAF and KRAS mutations have equivalent effects on tumorigenesis [44-46]. Although the possibility that other downstream targets of BRAF are mutated in conventional high-grade ovarian carcinomas cannot be completely ruled out, it would appear that the development of high grade serous carcinomas involves a pathway distinct from the MAP kinase signaling pathway.

In examining the relationship between PI3K/AKT and MAPK pathways, we found that *PIK3CA *amplification was the single most common genetic alteration in our Middle Eastern EOC cases and was mutually exclusive with gene mutations in both pathways (PIK3CA, KRAS, and BRAF). This represents strong genetic evidence that *PIK3CA *amplification possesses similar oncogenic function as the classical gene mutations in these pathways in ovarian cancer pathogenesis. The collective prevalence of genetic alterations (PIK3CA amplification, mutation, PTEN protein loss) in the PI3K/Akt pathway was particularly high in Middle Eastern EOC (60%), suggesting a significant role of the PI3K/Akt pathway in the pathogenesis of EOC.

## Conclusion

Our results showed that though mutation of PIK3CA is not a common event in the EOCs, its amplification is very common and may be a novel mechanism in activating the PI3K/AKT pathway in some EOCs. High prevalence of PI3K/AKT genetic alterations along with a low incidence of KRAS and BRAF mutations in Middle Eastern ovarian carcinomas, mutual exclusivity among genetic alterations in PI3K/AKT and MAPK pathways, support the notion that dysregulated PI3K/AKT pathways play an important role in the pathogenesis of ovarian cancers regardless of ethnic background. Understanding the alternative molecular pathways that result in epithelial ovarian cancers will allow specific biological targeting for therapy and prevention of ovarian cancer.

## Competing interests

The authors declare that they have no competing interests.

## Authors' contributions

JA carried out all the mutational analysis, helped in the project planning, as well as interpretation of data and contributed to the draft of the manuscript. PB contributed to the performance of immunohistochemistry, statstical analysis, and writing of the manuscript. WH carried out most of the PCR optimization and sequencing. ZJ conducted FISH analysis and real-time PCR validation. MS conducted some of the mutation analysis. AM helped in collecting the samples and provided us with the clinical data. SU participated in experimental design and writing of the manuscript. KSA conceived of the study, coordinated the study, and contributed to the draft of the manuscript.
